# Phytochemical investigation and hair growth studies on the rhizomes of *Nardostachys jatamansi* DC

**DOI:** 10.4103/0973-1296.80674

**Published:** 2011

**Authors:** Venkateswara Rao Gottumukkala, Tiruganasambandham Annamalai, Triptikumar Mukhopadhyay

**Affiliations:** *M/s. CavinKare Research Centre, 12, Poonamalle Road, Ekkattuthangal, Chennai-600 032, India*

**Keywords:** Hair growth, jatamansic acid, nardin, *Nardostachys jatamansi*, rhizomes

## Abstract

*Nardostachys jatamansi* DC rhizomes were subjected to extraction, fractionation, and isolation of terpenoid compounds. Three terpenoid compounds were isolated which are nardal, jatamansic acid, and nardin. These compounds were identified based on physical and spectral data (UV, IR,^1^H and^13^C NMR, 2D NMR, Mass) and comparison with authentic compounds. The crude extract, fractions, and two of the isolated compounds were tested for their hair growth activity. The hair growth studies showed good activities for the extract, fraction, and the isolated compounds.

## INTRODUCTION

*Nardostachys jatamansi* DC (Valerianaceae) is commonly called jatamansi or spikenard in English and it is a small shrub. The rhizomes of the plant were used since antiquity in the indigenous systems of medicine. The rhizomes and roots of plant are used as antistress agents in traditional medicine and marketed in India as an anticonvulsant Ayurvedic drug, Ayush 56.[1] The rhizome is being used as an aromatic adjunct in the preparation of medicinal oils, to promote growth of hair, and also imparts blackness.[2] The essential oil obtained from the roots of jatamansi showed fungi toxic activity,[3] antimicrobial,[4] antifungal,[5] hypotensive,[6] antiarrhythmic,[7] and anticonvulsant activity.[7,8] The rhizomes extracted with 50% ethanol showed hepatoprotective,[9] hypolipidemic,[10] and antiarrhythmic activity.[11] Previous reports on this plant occurring in different regions yielded terpenoids,[12] alkaloids,[13] neolignans and lignans,[14] coumarins,[1,15] and other compounds.[16–18] A few biologically active compounds were also reported. These are BR606 as bone sorption inhibitor for the treatment of osteoporosis and hypercalcemia,[19] jatamansone (valeranone) as hypotensive and tranquilizing agent,[20] antiarrhythmic and anticonvulsant agent.[7] Present study was therefore aimed to isolate and report the chemical constituents present in the hair growth active extract and to study their property.

## MATERIALS AND METHODS

### Plant material

The rhizomes of *N. jatamansi* were collected from bazaar in December, 2008 and a voucher specimen was deposited in M/s. CavinKare Research Centre, Chennai.

### General

Melting points reported are uncorrected. UV spectra were recorded on Shimadzu UV spectrophotometer. IR spectra were recorded on a Shimadzu IR prestige 21.^1^H NMR and ^13^C NMR spectra were recorded on a Bruker AMX 400 in CDCl_3_ with TMS as an internal standard and the chemical shifts being represented in parts per million (ppm, δ values). ESI mass spectrum was recorded on a Jeol SX 102/DA 6000 mass spectrometer. Column chromatography was performed on silica gel (100-200 mesh, Acme synthetic chemicals, Mumbai, India). Fractions and purity of the compounds were monitored by analytical thin-layer chromatography (TLC) and the spots were visualized by exposure to iodine vapor or spraying with 2,4-DNP solution or 5% sulphuric acid in methanol, followed by heating the plate at 110°C for 5 minutes. The TLC was performed on precoated silica gel plates (aluminium sheets 20 × 20 cm, silica gel 60 F_254_ plates of Merck KGaA, Germany). All solvents and reagents used were of analytical grade obtained from Merck.

### Extraction and isolation

The air-dried and finely powdered rhizomes (3.2 kg) were extracted with hexane through soxhlet apparatus for 8 hours. The extract was filtered and evaporated to dryness in vacuo using a rotary evaporator at 40°C to provide crude hexane extract (109 g). The crude hexane extract was subjected to vacuum liquid chromatography eluted with hexane : chloroform (9 : 1, 1 : 1) and chloroform to get corresponding fractions 25.91 g (Fr. I), 20.52 g (Fr. II), and 55.89 g (Fr. III), respectively. The fractions I and II showed hair growth promotion activity. Part of the fraction II, 8.21 g was subjected to silica gel column chromatography eluted with hexane : chloroform (isocratic, 60 : 40) to yield total of six subfractions, monitored by TLC. Compound 1 (800 mg) was obtained from subfraction 1 eluted with hexane : chloroform (7 : 3) after repeated silica gel chromatography. Compound 2 (69 mg) was obtained from subfraction 3 as colorless solid which was further crystallized in methanol to afford crystalline powder. Compound 3 (41 mg) came as colorless solid from subfraction 5 after passing through silica gel column, which was crystallized with hexane : methanol to obtain colorless crystals.

The isolated compounds were characterized by physical and spectral data and comparison with previously reported literature and additionally, compounds 1 and 3 were further confirmed with authentic samples by co-TLC.

**Compound 1**: Nardal: colorless oil;^1^H NMR (400 MHz, CDCl_3_): δ 9.38 (1H, s, CHO), 6.73 (1H, dq, 3-H), 3.73 (1H, dd, *J* = 4.4, 9.7 Hz, 3’-H), 1.79 (3H, d, *J* = 1.3 Hz, 2-CH_3_), 1.64 (3H, s, 9’-CH_3_), 0.79 (3H, d, *J* = 7.0 Hz, 5’-CH_3_).

**Compound 2**: Jatamansic acid: Colorless crystals, mp: 123-27°C,^1^ H NMR (400 MHz, CDCl_3_): δ 0.97 (6H, br, 10 and 11-H), 1.05 (3H, d, *J* = 6.2 Hz, 12-H), 2.43 (1H, septet, 9-H), 3.04 (1H, m, 8a-H), 5.75 (1H, d, *J* = 6.9 Hz, 6-H), 7.16 (1H, d, *J* = 6.0 Hz, 7-H).

**Compound 3**: Nardin: Colorless crystals, mp: 133-34°C,^1^ H NMR (400 MHz, CDCl_3_): δ 7.19 (1H, d, *J* = 9.9 Hz, 3-H), 3.55 (1H, m, 3’-H), 1.89 (3H, s, 2-CH_3_), 1.64 (3H, s, 9’-CH_3_), 0.79 (3H, d, *J* = 7.0 Hz, 5’-CH_3_).

### Hair growth promotion activity

The hair growth promotion activity was studied by using *in vivo* animal model.[21 22]

### Animals

Female Wistar rats weighing 120 to 150 g, from Dr. MGR Janaki College, Chennai, were used for hair growth study. Based on the guidelines of the ethical committee of the college, the animals were maintained in a clean cage and were provided with food and water *ad libitum*. The floor mat husk in each cage was removed and laid afresh on daily basis.

### Hair growth activity *in vivo*

The hair on the dorsal portion of the body of each animal was depilated using a standard, commercially available depilatory cream. After removal of the hair, the skin was cleaned with distilled water and wiped with surgical spirit. Four centimeter square area in the depilated dorsal skin was marked with permanent ink marker. The animals which showed any skin irritant response to the depilatory were removed from the experiment and replaced with new animal.

The rats were divided into 3 groups of 6 animals each. Group 1 animals served as negative control without any treatment. The negative control comprised of the vehicle for application (only) without having any active extract/fraction/compound. Group 2 animals were applied 50 µl of commercial 2% Minoxidil solution in the predefined area. The group 3 animals were applied samples (extract/fractions/compounds) prepared in liquid paraffin at 2%. The quantity of the solution used for the experiment was 50 µl per 4 cm sq area per animal. The application of the Minoxidil and the test samples were continued for 30 days. The observations such as hair growth initiation time (HGIT) in days and hair growth completion time (HGCT) in days were recorded for all the animals on daily basis. The HGIT was defined as the presence of new hair in the treated site of 4 cm sq area. The HGCT was defined as complete filling of hair in the treated site of 4 cm sq area in each animal which become indistinguishable from the adjacent untreated portion of the body. The average of HGIT and HGCT was calculated for each group along with control animals. The untreated control for HGIT is 10 days and HGCT is 30 days. The percentage reduction in HGCT (% reduction in HGCT) for the treatment is calculated by the formula given below. The results of hair growth activity are shown in Table 1.

**Table 1:** Comparison of hair growth results

Extract/fraction/ compound	Hair growth initiation time (HGIT in days)	Hair growth completion time (HGCT in days)	% Reduction in time
Hexane extract	9	20	30
Fraction I	9	20	30
Fraction II	9	20	30
Nardin	10	22	26.67
Jatamansic acid	9	23	23.33
Minoxidil	5	17	43.33
Untreated control	10	30	0

$$\mathrm{Calculation}\;=\;\frac{HGCT\;in\;untreated\;control\;–\;HGCT\;in\;test\;sample}{HGCT\;in\;untreated\;control}\;\times \;100$$

## RESULTS AND DISCUSSIONS

**Figure 1:**
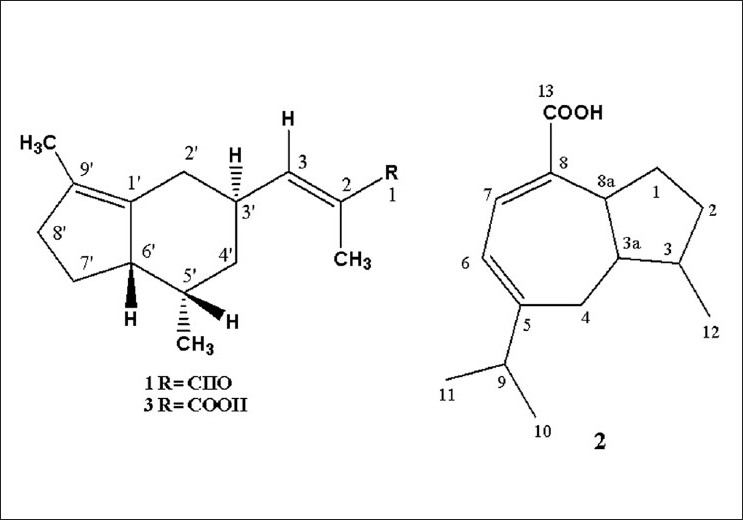
Compounds from *N. jatamansi* DC

The preliminary screening of the hexane extract of the rhizomes of *N. jatamansi* showed positive response in hair growth promotion activity. The bioassay-guided purification of the hexane fractions of the rhizomes of *N. jatamansi* repeated chromatography with a silica gel and recrystallization with solvents furnished nardal, jatamansic acid, and nardin [Figure 1]. The structure of the compounds were elucidated on the basis of UV, IR,^1^H and ^13^C NMR and Mass spectral dataand comparison with an authentic samples.

The hair growth promotion activity of nardin and jatamansic acid showed moderate reduction in hair growth time, whereas minoxidil, a positive control showed excellent activity in the standard method but it had other side effects.[22] Even though the plant is being used in the preparation of hair oils, so far there are no reports on the compounds responsible for hair growth promotion activity.

The compound 1 was readily recognized as sesquiterpene aldehyde by its preliminary spectral data. Its proton spectrum showed the presence of an aldehyde group δ 9.38 (s) in the molecule. The proton spectrum further showed one secondary methyl at δ 0.79 (d, *J* = 7.0 Hz), two double-bonded methyls at δ 1.64 (s) and 1.79 (d, *J* = 1.3 Hz) and an olefinic proton at δ 6.73 as double quartet. Its carbon spectrum showed the presence of 15 signals. It showed an aldehydic carbonyl signal at δ 195.8 and a double-bonded methyl carbon at δ 9.2, which is connected to the a-carbon of the α,β-unsaturated system. Furthermore, the spectrum showed four signals at δ155.9, 137.4, 132.5, and 131.9 which correspond to two double bonds in the molecule. By comparing the spectral data of the compound 1 [Table 2] well corroborated with previously reported values as well as by co-TLC with an authentic sample and established as nardal.[23]

**Table 2:** Comparison of ^1^H and ^13^C NMR spectral data of compounds 1 and 3 in CDCL_3_ (400 MHz for ^1^H and 100 MHz for ^13^C NMR)

Carbon	1 (δ_H_)	1 (δ_C_)	3 (δ_H_)	3 (δ_C_)
1	9.38 s	195.8	-	173.9
2	-	137.4	-	133.3
3	6.73dq	155.9	7.19 d (9.9)	146.3
1’	-	132.5	-	131.2
2’	2.02 m, 2.20 m	37.4	1.99 m, 2.20 m	37.5
3’	3.73 dd (4.4, 9.7)	47.5	3.55 m	47.5
4’	1.82 -1.92 br m	25.4	1.75 - 1.85 br m	25.4
5’	1.82 -1.92 br m	32.9	1.75 - 1.85 br m	33.1
6’	1.82 -1.92 br m	34.6	1.75 - 1.85 br m	34.6
7’	1.43 m	24.5	1.43 m	24.6
8’	2.20 m, 2.95 m	28.8	2.20 m, 2.94 m	28.8
9’	-	131.9	-	125.3
2-CH_3_	1.79 d (1.3)	9.2	1.89 s	13.5
5’-CH_3_	0.79 d (7.0)	12.0	0.79 d (7.0)	12.0
9’-CH_3_	1.64 s	13.5	1.64 s	12.1

J values (Hz) are given in parentheses

The compound 2 was identified as sesquiterpene acid based on its preliminary spectral data. The proton spectrum showed the presence of carboxylic peak at δ 12.0 (1H, br) in the molecule. It also showed an isopropyl methyl group at δ 0.97 as broad singlet, one secondary methyl at δ 1.05 (d, *J* = 6.2 Hz), and two olefinic protons at 5.75 (d, *J* = 6.9 Hz) and 7.16 (d, *J* = 6.0 Hz). The olefinic protons are connected to β- and γ-protons of conjugated carboxylic acid system. Its carbon spectrum showed the presence of 15 signals, including one carboxylic acid signal at δ 175.0, four olefinic signals at δ 160.6, 135.3, 134.4, and 117.0. By revealing the literature, the spectral data [Table 3] of the compound 2 is exactly matching with those of previously reported values. So, the compound has been identified as jatamansic acid.[24]

**Table 3:** ^1^H and ^13^C NMR spectral data of compound 2 in CDCL_3_ (400 MHz for ^1^H NMR and 100 MHz for ^13^C NMR)

Carbon	δ_H_	δ_C_
1	1.50m, 2.45 m	33.0
2	1.10 m	28.6
3	1.22 m	38.5
3a	1.70 m	43.6
4	1.78 m	26.1
5	-	160.6
6	5.75 d (6.9)	117.0
7	7.16 d (6.0)	135.3
8	-	134.4
8a	3.04 m	45.7
9	2.43 m	38.5
10	0.97 br s	21.5
11	0.97 br s	21.7
12	1.05 d (6.2)	16.1
13	~ 12.00 br	175.0

J values are given in parentheses

The compound 3 was readily recognized as sesquiterpene acid by its preliminary spectral data. The proton spectrum showed one secondary methyl at δ 0.79 (d, *J* = 7.0 Hz), two double-bonded methyls at δ 1.64 (s) and 1.79 (d, *J* = 1.3 Hz), and an olefinic proton at δ 7.19 (d, *J* = 9.9 Hz). Its carbon spectrum showed the presence of 15 signals. It showed carboxylic acid signal at δ 173.9, a double-bonded methyl carbon at δ 13.5, and four olefinic carbons at δ 146.3, 133.3, 131.2, and 125.3. All the spectral data of the compound 3 [Table 2] were found to be identical to those reported for nardin.[23] The identification of the compound was further confirmed by direct comparison with reference sample through mixed melting point and co-TLC. The compound 3 is an oxidized form of nardal.

The results of hair growth promotion [Table 1] showed that crude hexane extract required less time than pure compounds, nardin and jatamansic acid. It is worth mentioning that many crude extracts or active fractions are showing better activity than individual compounds.

## CONCLUSION

To our best knowledge, the present study is the first report of the isolation of active compounds from *N. jatamansi* for hair growth studies. Additionally, even though the plant has been worked out very well for the past 70 years, this is the first time all three compounds were reported in same isolation.

## References

[1] Chatterjee A, Basak B, Datta U, Banerji J, Neuman A, Prange T (2005). Studies on the chemical constituents of *N.jatamansi* DC (Valerianaceae). Indain J Chem Br.

[2] Kirthikar KR, Basu BD, Mahendra Pal Singh BS (1993). Indian Medicinal Plants.

[3] Mishra D, Chaturvedi RV, Tripathi SC (1995). The fungitoxic effect of the essential oil of the herb *Nardostachys jatamsnsi* DC. Trop Agriculture (Trinidad).

[4] Rao JT (1986). Antimicrobial properties of the essential oil of *Nardostachys jatamansi*. PAFAI Jr.

[5] Girgune JB, Jain NK, Garg BD (1978). Antifungal activity of some essential oils, 2. Indian Drugs.

[6] Arora RB, Singh KP, Das PK, Mistry PN (1958). Prolonged hypotensive effect of the essential of *N.jatamansi*. Arch Intern Pharma Ther.

[7] Arora RB, Sharma PL, Kapila K (1958). Anti-arrhythmic and anticonvulsant activity of jatamansone. Indian J Med Res.

[8] Rao VS, Rao A, Karanth KS (2005). Anticonvulsant and neurotoxicity profile of *Nardostachys jatamansi*. J Ethnopharmacol.

[9] Ali S, Ansari KA, Jafry MA, Kabeer H, Diwakar G (2000). *Nardostachys jatamansi* protects against liver damage induced by thioacetamide in rats. J Ethnopharmacol.

[10] Dixit VP, Jain P, Joshi SC (1988). Hypolipidaemic effects of *Curcuma longa* Linn., and *Nardostachys jatamansi* DC, in triton-induced hyperlipidaemic rats. Indian J Physiol Pharmacol.

[11] Arora RB, Madan BR (1956). Antiarrhythmics. III. Antiarrhythmic activity of Nardostachys jatamansi (an Indian indigenous drug). Indain J Med Res.

[12] Mahalwal VS, Ali M (2002). Volatile constituents of the rhizomes of *N.jatamansi* DC. J Essn Oil-bearing Plants.

[13] Hirose Y, Yonemitsu K, Sonoda T (1978). Chemical studies on the components of *N.jatamansi* de Candolle I. Shoyakugaku Zasshi.

[14] Bagchi A, Oshima Y, Hikino H (1991). Neolignans and lignans of *N.jatamansi* roots. Planta Medica.

[15] Shanbhag SN, Mesta CK, Maheswari ML, Paknikar SK, Bhattacharyya SC (1964). Terpenoids. LII. Jatamansin, a new terpenic coumarin from *N. jatamansi*. Tetrahedron.

[16] Sastry SD, Maheswari ML, Chakravarti KK, Bhattacharya SC (1967). Chemical constituents of *N. jatamansi*. Perfumery Essential Oil Record.

[17] Sastry SD, Maheswari ML, Chakaravarthi KK, Bhattacharya SC (1967). Terpenoids. CVI. The structure of calerenol. Tetrahedron.

[18] Singh V, Ali M (2003). New phytoconstituents from *N.jatamansi* rhizomes. J Saudi Chem Soc.

[19] Kawashima A, Kishimoto M, Morimoto S, Akyama T, Maejima A, Kawada K (1996). Extraction of novel compound BR-606 from *N.jatamansi* roots as bone sorption inhibitor for treatment of osteoporosis and hypercalcemia. Jpn Kokai Tokkyo Koho.

[20] Arora RB, Arora CK (1963). Hypotensive and tranquilizing activity of jatamansone (valeranone), a sesquiterpene from N. jatamansi.Proc. Intl Pharmacol Meeting 2^nd^ Prague.

[21] Adirajan N, Ravikumar T, Shanmugasundaram N, Mary B (2003). *In vivo* and *in vitro* evaluation of hair growth potential of *Hibiscuss rosa-sinensis* Linn. J Ethnopharmacol.

[22] Semalty M, Semalty A, Joshi GP, Rawat MS (2010). Development and *in vivo* studies of herbal hair oil for hair growth promotion. Indian Drugs.

[23] Rao GV, Annamalai T, Mukhopadhyay T (2008). Nardal, a new sesquiterpene aldehyde from the plant, Nardostachys jatamansi DC. Indian J Chem Br.

[24] Rucker G, Panikar SK, Mayer R, Breitmaier E, Will G, Wiehl L (1993). Revised structure and stereochemistry of Jatamansic acid. Phytochemistry.

